# Rapid Geographic Expansion, Decelerating Growth, and Six-Month Scenario Projections of Bundibugyo Virus Disease in the Democratic Republic of the Congo: Analysis of Situation Reports 1–61

**DOI:** 10.3390/v18101104

**Published:** 2026-10-06

**Authors:** David Lupande Mwenebitu, Demelash Areda, Pascal Adroba, Franklin Mwamba, Philippe Boribo Kikunda, Olivier Mukuku, Danstan Bagenda, Misaki Wayengera, Patrick D. M. Katoto, Gaylord Ngaboyeka

**Affiliations:** 1 Laboratoire de Recherche Biomédicale Prof. Lurhuma, Faculty of Medicine, Université Catholique de Bukavu, B.P. 285, Bukavu, South Kivu, Democratic Republic of the Congo; katoto.chimusa@ucbukavu.ac.cd; 2 Center for Tropical Diseases and Global Health, Université Catholique de Bukavu, B.P. 285, Bukavu, South Kivu, Democratic Republic of the Congo; 3 Department of Biology, Institut Supérieur Pédagogique de Baraka (ISP Baraka), Baraka, South Kivu, Democratic Republic of the Congo; 4 Interdisciplinary Consortium for Epidemics Research (ICER)–Regional Network, Bukavu, South Kivu, Democratic Republic of the Congo; 5 College of Arts and Sciences, Ottawa University, 15950 N Civic Center Plaza, Surprise, AZ 85374, USA; demelash.biffa@gmail.com; 6 Laboratoire Provincial de Santé Publique de l’Ituri, Bunia, Ituri, Democratic Republic of the Congo; pascaladroba2017@gmail.com; 7 Institute for Health Sciences Research (IRSS), B.P. 10.183, Kinshasa, Democratic Republic of the Congo; franklinmwamba67@gmail.com; 8 Department of Mathematics, Statistics and Computer Science, Faculty of Sciences and Technology, University of Kinshasa, B.P. 190, Kinshasa, Democratic Republic of the Congo; 9 Faculty of Science and Technology, Université Catholique de Bukavu, B.P. 285, Bukavu, South Kivu, Democratic Republic of the Congo; kikunda.boribo@ucbukavu.ac.cd; 10 Department of Management Information Systems, Institut Supérieur Pédagogique de Bukavu (ISP-Bukavu), B.P. 854, Bukavu, South Kivu, Democratic Republic of the Congo; 11 École de Santé Publique, Université Officielle de Goma, B.P. 204, Goma, North Kivu, Democratic Republic of the Congo; oliviermukuku@yahoo.fr; 12 Department of Anesthesiology, University of Nebraska Medical Center, 42nd and Emile Street, Omaha, NE 68198, USA; dbagenda@mac.com; 13 College of Health Sciences, Makerere University, Kampala P.O. Box 7062, Uganda; waymisaki@gmail.com; 14 École Régionale de Santé Publique, Université Catholique de Bukavu, B.P. 285, Bukavu, South Kivu, Democratic Republic of the Congo; amaningaboyekagaylord@gmail.com

**Keywords:** Bundibugyo virus disease, Ebola, Democratic Republic of the Congo, epidemic growth, doubling time, case fatality ratio, geographic spread, scenario projections

## Abstract

In May 2026, the Democratic Republic of the Congo declared an outbreak of Bundibugyo virus disease caused by *Bundibugyo ebolavirus*. We analysed aggregated official Situation Reports (SitReps) issued from 14 May through 14 July 2026 to describe the reported epidemic trajectory, geographic expansion, mortality, interval-specific growth, and conditional six-month projections. Confirmed cases increased from 8 to 2073 and confirmed deaths reached 796; 45 health zones in five provinces were affected. Average interval-specific growth rates generally declined over the observation period, although cumulative cases and the geographic footprint continued to increase. Gompertz and Richards curves described the observed cumulative trajectory better than logistic and exponential curves according to root mean square error (RMSE), the corrected Akaike information criterion (AICc), and the Bayesian information criterion (BIC); this comparison reflects in-sample fit only. Conditional six-month scenarios varied substantially across assumptions (approximately 2435 to 10,839 cumulative cases by 14 January 2027) and are contingent and highly uncertain; Gompertz fits showed underprediction of the subsequently reported total in four temporal checks. Reported case fatality ratios (CFRs) varied across provinces but are vulnerable to outcome delay, incomplete case ascertainment, small denominators, and retrospective reclassification. The results support continued surveillance and response planning but do not identify the causes of epidemic slowing or establish that transmission was controlled.

## 1. Introduction

Ebola virus disease (EVD) is a severe viral haemorrhagic fever caused by viruses belonging to the genus *Orthoebolavirus*, family *Filoviridae*, and order *Mononegavirales*. Among the recognised orthoebolaviruses associated with human disease, *Bundibugyo ebolavirus* represents one of the least extensively characterised species because relatively few outbreaks and clinical datasets have been documented [1]. Bundibugyo virus disease was first recognised during an outbreak in western Uganda in 2007 and was subsequently reported in the Democratic Republic of the Congo (DRC) in 2012 [2]. Compared with *Zaire ebolavirus*, the principal cause of previous large DRC outbreaks, Bundibugyo virus remains associated with major gaps in available medical countermeasures. Vaccines and therapeutics developed primarily against *Zaire ebolavirus*, including the recombinant vesicular stomatitis virus–based *Zaire ebolavirus* vaccine (rVSV-ZEBOV) and Zaire-specific monoclonal antibody products, cannot be assumed to provide equivalent protection against Bundibugyo virus because of substantial antigenic differences between species [3].

Taxonomically, Bundibugyo virus (BDBV) belongs to the species *Bundibugyo orthoebolavirus*. It is an enveloped, non-segmented, negative-sense single-stranded RNA virus with a genome of approximately 19 kilobases (kb) encoding seven major structural and functional proteins: nucleoprotein (NP), viral proteins (VPs) 35 and 40, glycoprotein (GP), VP30, VP24, and the RNA-dependent RNA polymerase (L) [4]. Comparative genomic studies have shown that Bundibugyo virus is genetically distinct from Zaire, Sudan, Taï Forest, and Reston orthoebolaviruses, particularly within the glycoprotein gene and other regions relevant to antigenicity, host-cell entry, immune evasion, and molecular diagnostics [5]. Whole-genome sequencing during an outbreak is therefore essential for confirming species identity, evaluating genetic relatedness to historical strains, distinguishing a new zoonotic introduction from sustained human-to-human transmission, and monitoring mutations that could affect diagnostic assay performance. Detailed phylogenetic data from the 2026 Bunia outbreak should be incorporated when officially released by the Institut National de Recherche Biomédicale (INRB), the World Health Organization (WHO), or other authorised genomic surveillance partners [6,7].

On 15 May 2026, the DRC Ministry of Health officially declared an EVD outbreak following laboratory confirmation of Bundibugyo virus in Ituri Province. The outbreak subsequently expanded across eastern and northeastern DRC, affecting Ituri, North Kivu, South Kivu, Haut-Uélé, and Tshopo. By 14 July 2026, national surveillance reports documented 2073 confirmed cases, 796 confirmed deaths, and transmission across 45 health zones. The rapid geographic expansion occurred in a setting characterised by recurrent insecurity, population displacement, mining-related mobility, cross-provincial travel, restricted access to healthcare, and variable availability of decentralised molecular diagnostics and treatment capacity [8,9].

Retrospective epidemiological investigations suggested that transmission may have preceded formal recognition by several weeks. Delayed detection may have been influenced by limited access to Bundibugyo-specific molecular assays in peripheral health zones, reliance on diagnostic platforms primarily configured for *Zaire ebolavirus*, incomplete community-based surveillance, and logistical barriers to specimen referral. Such delays are particularly consequential for EVD because late isolation, delayed contact identification, community deaths, and undetected transmission chains can accelerate geographic dissemination and increase mortality [10,11].

This study synthesised official situation reports (SitReps) issued between 14 May and 14 July 2026. The objectives were to describe the reported epidemic trajectory and geographic spread; estimate interval-specific average growth rates and doubling times; compare candidate cumulative growth models; summarise provincial burden and reported case fatality ratios (CFRs); develop conditional six-month planning scenarios; and assess forecast performance using limited temporal validation. Because the available reports contained aggregated surveillance counts, the study did not attempt to estimate intervention effects, transmission mechanisms, or individual-level risk factors.

## 2. Materials and Methods

### 2.1. Data Sources

Primary data were derived from 53 available official SitReps numbered 1–61 and issued by the Centre des Opérations d’Urgence de Santé Publique (COUSP-RDC) of the Institut National de Santé Publique (INSP), covering 14 May to 14 July 2026 [12]. Eight of the 61 sequentially numbered SitReps (Nos. 3, 4, 8, 9, 12, 29, 43 and 45) were unavailable to the authors at the time of extraction. Analyses used the reporting dates and observations in the available reports without interpolation. The available files did not document why the eight reports were unavailable. Supplementary contextual information was drawn, where necessary, from official national, WHO, and United Nations communications. INSP/COUSP reports remained the primary source for quantitative analyses.

### 2.2. Case Definitions

Confirmed cases: Any person with a positive BDBV nucleic acid amplification test (NAAT) performed by the INRB reference laboratory, including its authorised mobile diagnostic units deployed in Ituri and in the other affected provinces.

Confirmed deaths: deaths in laboratory-confirmed BDBV cases.

Case fatality ratio (CFR) = confirmed deaths/confirmed cases × 100%.

### 2.3. Statistical Analysis

Each available SitRep reporting date was treated as an observation point. Descriptive analyses included cumulative confirmed cases and deaths, recoveries, suspected cases, patients in isolation, contact follow-up, and the numbers of affected provinces and health zones when these indicators were reported. Changes between reports were calculated from the reported totals. Because cumulative totals were sometimes revised during District Health Information Software 2 (DHIS2) cleaning, harmonisation, and reclassification, differences between successive reports were not interpreted automatically as incident cases. The available extracted series did not contain a revision log that permitted the magnitude or direction of historical revisions to be quantified.

To characterise change in reported epidemic growth without imposing discrete epidemic phases, the average growth rate between consecutive available observations was calculated as *r_i_* = [ln(*y_i_*_+1_) − ln(*y_i_*)]/Δ*t_i_*, where *y_i_* and *y_i_*_+1_ are cumulative confirmed-case counts at successive reporting dates and Δ*t_i_* is the number of days between them. The corresponding doubling time was *T*_d,*i*_ = ln(2)/*r_i_* when *r_i_* was positive. These are interval-specific descriptive rates, not instantaneous transmission-rate estimates. They are affected by serial correlation, reporting delay, changing ascertainment, and retrospective revision of cumulative totals.

Province- and health-zone-level burden was summarised using cumulative confirmed cases, confirmed deaths, reported CFRs, and proportional contribution to the national total. Geographic expansion was described using the number of affected provinces and health zones. Reported CFR was calculated as cumulative confirmed deaths divided by cumulative confirmed cases multiplied by 100. Wilson score 95% confidence intervals were calculated to show sampling imprecision [13]. These intervals do not correct for delayed outcomes, preferential ascertainment of severe or fatal cases, missed mild cases, changing surveillance sensitivity, or retrospective reclassification.

Four cumulative growth functions were examined because they represent progressively more flexible descriptions of cumulative epidemic growth [14,15]. The exponential model, C(t) = a exp(bt), assumes constant proportional growth with no saturation and was retained only as an early-growth benchmark/comparator; it was not treated as a plausible full-trajectory forecast after clear deceleration. The logistic model, C(t) = K/{1 + exp[−r(t − *t*_0_)]}, assumes approximately symmetric acceleration and deceleration around an inflection point. The Gompertz model, C(t) = K exp[−b exp(−rt)], allows asymmetric growth, with earlier deceleration and a longer approach to the upper asymptote. The Richards model generalises the logistic curve by adding a shape parameter, allowing the degree of asymmetry and location of the inflection to be estimated more flexibly. In these expressions, C(t) is cumulative cases at time t; K is the asymptotic cumulative total; r is a growth/deceleration parameter; a is the exponential scale parameter; b is the Gompertz displacement parameter; *t*_0_ is the logistic/Richards inflection-time parameter; and the Richards shape parameter s controls asymmetry. Models were fitted by nonlinear least squares (SciPy curve_fit) to the same 21-observation reconciled national series (the Reconciled Analytical Series and Data Lineage subsection; Supplementary Table S1), with time indexed as t = SitRep number (t = 61 on 14 July 2026), and compared using root mean square error (RMSE), the corrected Akaike information criterion (AICc) [16], and the Bayesian information criterion (BIC) [17]. AICc and BIC were computed from the Gaussian log-likelihood, counting the residual variance as an additional parameter. The Richards shape parameter was constrained to s ≥ 0.01 to exclude biologically implausible solutions. Model selection was interpreted jointly with epidemiological plausibility and visual agreement between observed and fitted trajectories.

#### Reconciled Analytical Series and Data Lineage

Of the 53 available SitReps, 21 were used for interval-specific growth-rate estimation and model fitting. The analytical series is the union of two sets of reports: (i) SitRep 1 and the last available SitRep in each consecutive block of five reports (SitReps 5, 10, 15, 20, 25, 30, 35, 40, 44, 50, 55 and 61), which form Table 1 (for this block the analytical period was truncated to SitReps 41–44, because SitRep 45 was unavailable at the time of analysis); and (ii) intermediate milestone reports at approximately four- to six-report spacing (SitReps 6, 14, 24, 34, 39, 49, 53 and 57), which are listed in Supplementary Table S1. Where the two sets overlapped (SitReps 1, 10, 20, 30, 44 and 61), the extracted values agreed exactly. This construction was not a pre-specified sampling rule, and the remaining 32 available SitReps were not used for model fitting; this is acknowledged as a limitation (Section 4.2).

Each SitRep was assigned the reporting date printed on the report. Because SitReps were issued daily, the time index was set to the SitRep number (t = 1, …, 61), and interval lengths Δ*t* were computed as differences in t. The 21 selected reports had distinct reporting dates, so no duplicate-date resolution was required. Each observation is the cumulative confirmed-case total as published in that SitRep; retrospective corrections published in later reports were not back-propagated to earlier observations. The resulting series was non-decreasing, so no downward revision had to be resolved. The 21 observations, with reporting dates, source SitRep numbers, time index, and the derived interval-specific growth rates and doubling times, are provided in Supplementary Table S1.

**Interval-specific growth-rate and doubling-time trajectory.** For successive observations, *r_i_* denotes the average daily proportional growth rate, *y_i_* and *y_i_*_+1_ denote the reported cumulative counts at the beginning and end of interval i, Δ*t_i_* denotes the interval length in days, and *T*_d,*i*_ denotes the doubling time implied by a positive *r_i_*. These estimates describe changes in the reported cumulative series and do not identify the causes of those changes.

Limited temporal validation was conducted by refitting the Gompertz model using data available through four progressively later truncation points (SitReps 39, 44, 50, and 55) and comparing each resulting projection with the reported SitRep 61 total. These origins were selected to span progressively longer observation windows while retaining enough observations for stable nonlinear fitting; earlier origins were dominated by sparse early counts and yielded poorly constrained saturation parameters. Because only four origins were evaluated, this analysis is explicitly presented as limited temporal validation rather than a comprehensive rolling-origin assessment. The four checks share the same target (SitRep 61) and overlapping training data, so they are not independent.

**Six-month scenario projections.** Three conditional scenarios were constructed from the 14 July 2026 forecast origin (t = 61), with horizons of 1 month (14 August 2026; t = 92), 3 months (14 October 2026; t = 153) and 6 months (14 January 2027; t = 245). The logistic saturation and Gompertz deceleration scenarios evaluate the fitted curves reported in Table 2 at these horizons, without refitting or re-anchoring to the observed SitRep 61 total; because both curves are bounded by their asymptote K, their central values cannot exceed the corresponding K in Table 2. Uncertainty for these two scenarios was summarised by a residual bootstrap: fitted residuals were resampled with replacement 2000 times, the model was refitted to each resampled series, and the 2.5th and 97.5th percentiles of the projected values were reported. The constant recent-increase scenario extrapolates linearly the mean reported absolute increase between SitRep 47 (30 June 2026; 1406 cases) and SitRep 61 (14 July 2026; 2073 cases), i.e., (2073 − 1406)/14 days = 47.6 cases per day; it is not model-based and has no probabilistic interval.

All statistical analyses and visualisations were performed using R version 4.3.3 (R Foundation for Statistical Computing, Vienna, Austria) and Python version 3.11.9 (Python Software Foundation, Wilmington, DE, USA), with the pandas (version 2.2.2), NumPy (version 1.26.4), SciPy (version 1.13.1), statsmodels (version 0.14.2), emcee (version 3.1.6) [18], and matplotlib (version 3.8.4) libraries. An exploratory Bayesian Gompertz fit with a negative-binomial observation model, implemented with the affine-invariant ensemble sampler in emcee [18] and cross-checked against an equivalent specification in brms [19], was used during analysis to confirm that the reported residual-bootstrap intervals were not artefacts of the least-squares assumption; it is not reported here because it did not alter any conclusion. A two-sided significance level of 0.05 was applied where inferential statistics were reported.

## 3. Results

### 3.1. Overall Epidemic Trajectory

**Figure 1:**
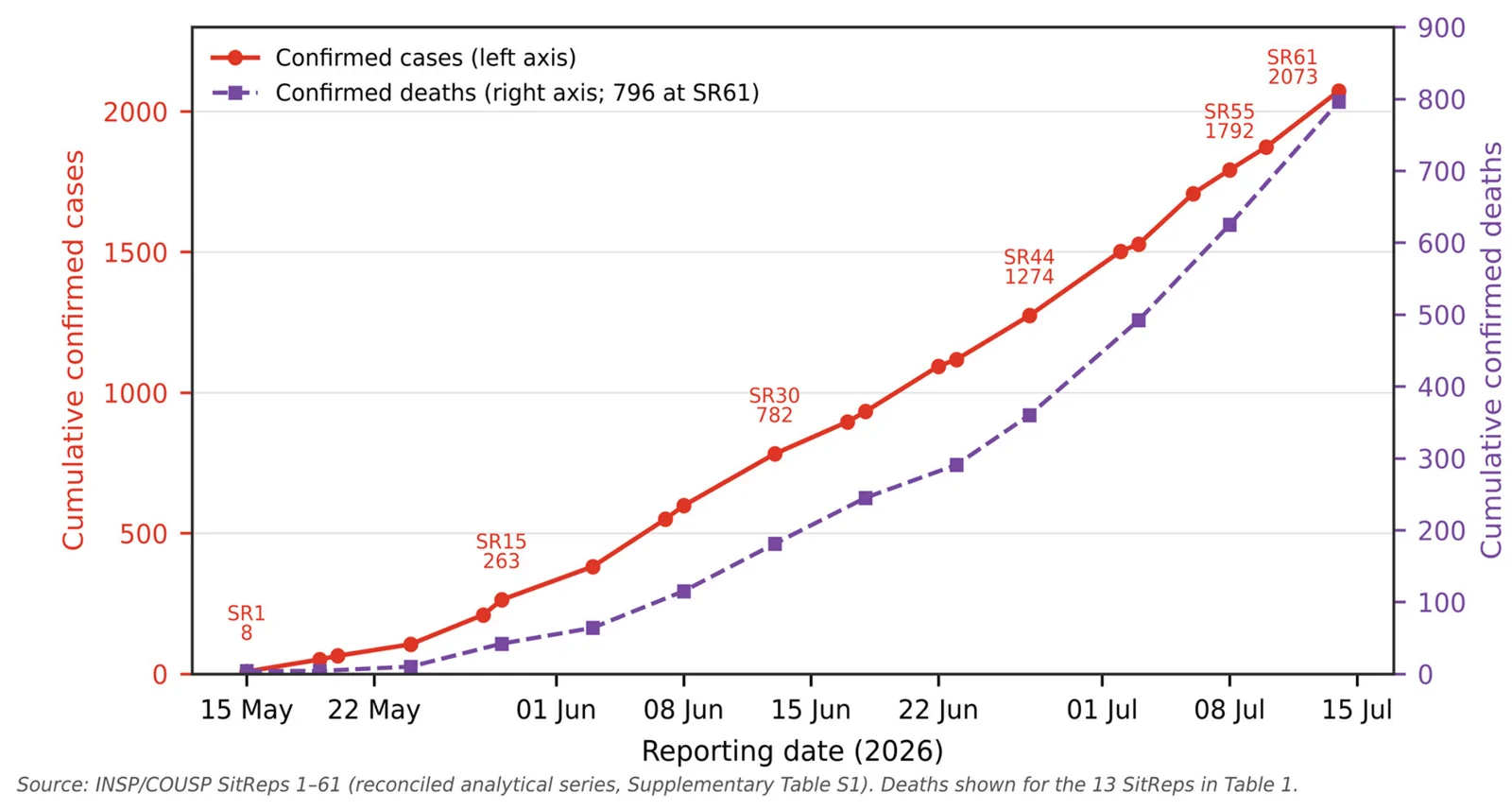
Reported cumulative confirmed cases and deaths, SitReps 1–61, Bundibugyo virus disease outbreak, Democratic Republic of the Congo, 14 May–14 July 2026. Cases are the 21 reconciled observations (Supplementary Table S1), ending at SitRep 61 (2073 cases); deaths are the 13 values reported in Table 1 (796 at SitRep 61). Source: INSP/COUSP.

The epidemic expanded rapidly during the observation period (Figure 1). Confirmed cases increased from 8 in SitRep 1 on 14 May 2026 to 2073 in SitRep 61 on 14 July 2026, representing a 259-fold increase over 61 days. During the same period, cumulative confirmed deaths increased from 4 to 796, while cumulative recoveries reached 377. The reported case fatality ratio increased from 16.0% in SitRep 15 on 29 May to 26.7% in SitRep 42 on 25 June, 31.2% in SitRep 47 on 30 June, and 38.4% in SitRep 61 on 14 July 2026. The number of affected health zones rose from 3 on 14 May to 22 by 29 May, 34 by 25 June, 37 by 6 July, and 45 by 14 July. The number of affected provinces increased from one to five following the recognition of Haut-Uélé and Tshopo in July 2026.

The reported trajectory remained upward through mid-July 2026. Cumulative cases, deaths, and the number of affected health zones and provinces continued to increase, while interval-specific proportional growth generally slowed. Because cumulative counts can increase through delayed confirmation and retrospective reclassification, these data alone do not quantify contemporaneous transmission intensity or demonstrate specific gaps in response performance.

### 3.2. Epidemic Growth Rate and Doubling Time

#### 3.2.1. Observed Growth Trajectory

**Figure 2:**
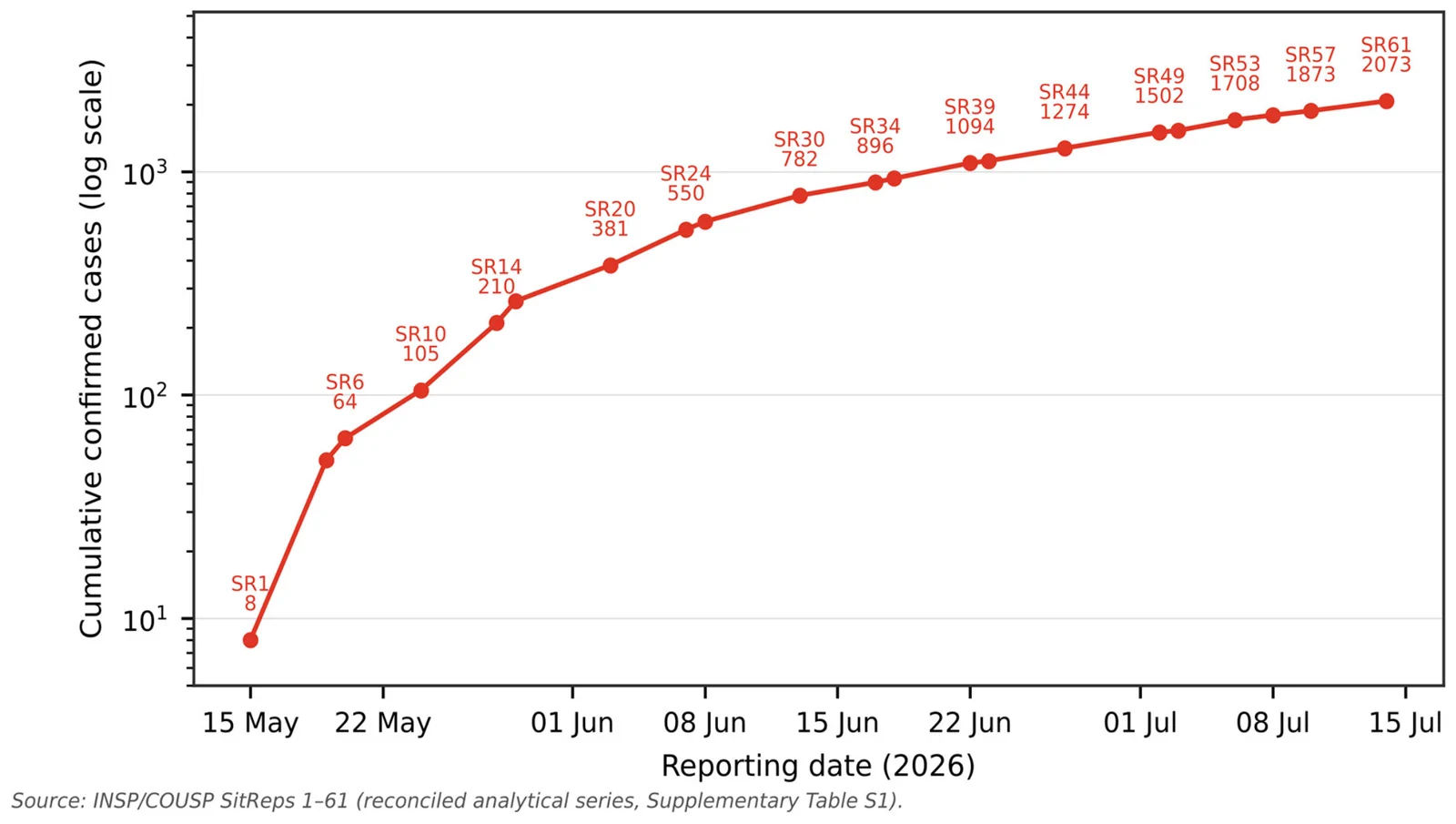
Reported cumulative confirmed cases on a logarithmic scale for the 21 reconciled observations, SitReps 1–61 (14 May–14 July 2026). Labels give the SitRep number and the cumulative count. Source: INSP/COUSP.

The cumulative confirmed-case series became progressively less steep over the observation period (Figure 2), while remaining upward through SitRep 61. This pattern is consistent with deceleration in proportional growth but does not define discrete epidemic phases or establish a sustained decline. Interval-specific estimates are presented in Section 3.2.3.

Reported cumulative cases increased from 1406 at SitRep 47 to 2073 at SitRep 61. Consequently, slower proportional growth should not be interpreted as proof of outbreak control. The aggregated data do not permit the observed slowing to be attributed to treatment-centre capacity, diagnostic expansion, contact tracing, other response measures, susceptible depletion, changes in ascertainment, or reporting practices.

The growth estimates should be interpreted as descriptive features of the reported cumulative trajectory. The observations were serially correlated and subject to reporting delays and retrospective cleaning, harmonisation, and reclassification. Because no revision log was available, the effect of historical revisions on the estimated rates could not be quantified.

The changing slope of the reported cumulative trajectory motivated the interval-specific analysis presented in Section 3.2.3; that analysis does not establish causal phases or intervention effects.

#### 3.2.2. National-Level Growth Model Comparison

**Figure 3:**
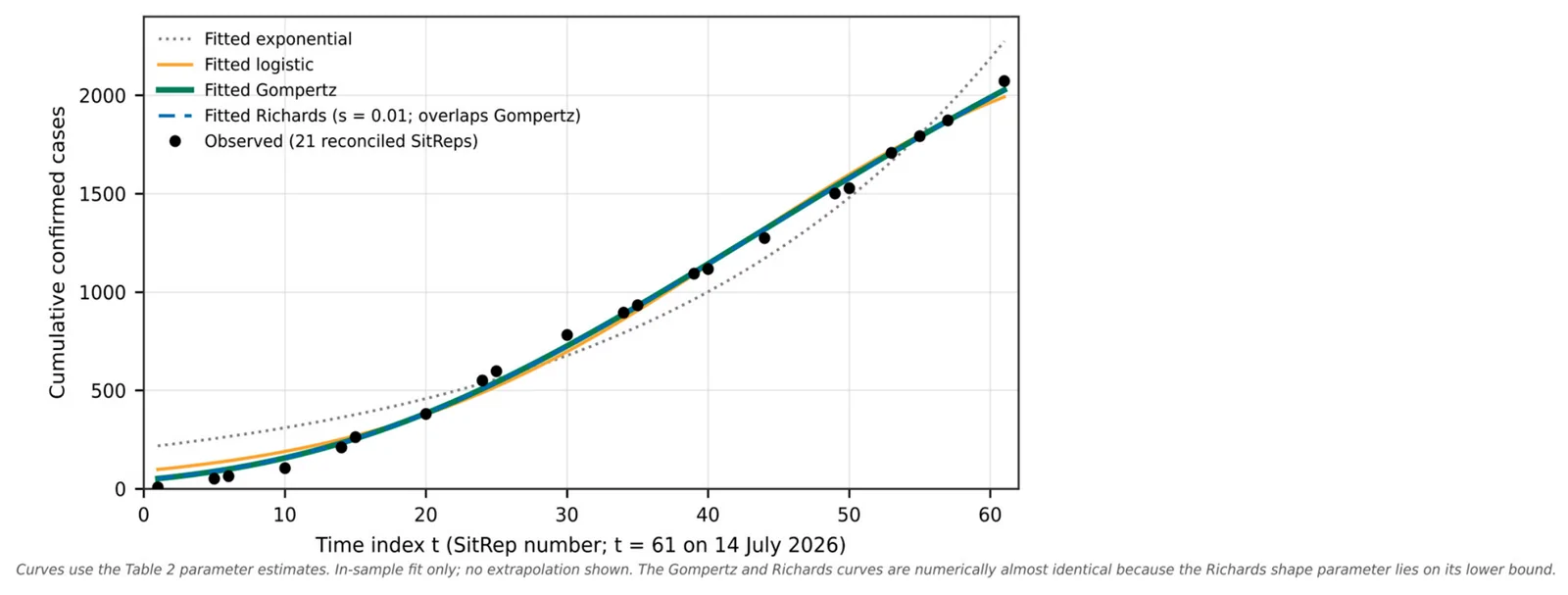
Observed cumulative confirmed cases (21 reconciled observations; black points) and in-sample fitted exponential, logistic, Gompertz and Richards curves (lines) using the parameter estimates in Table 2. The Richards curve is drawn as a dashed overlay because it is numerically almost identical to the Gompertz curve, the Richards shape parameter lying on its lower bound. No extrapolation beyond SitRep 61 is shown.

Four candidate nonlinear growth models (exponential, logistic, Gompertz, and generalised-logistic (Richards)) were fitted to the reconciled 21-point national aggregated case series and compared by RMSE, AICc, and BIC (Section 2.3; Figure 3). The Gompertz model provided the best fit by both AICc and BIC, followed by the Richards model, the logistic model, and, considerably worse, the unbounded exponential model, which is not biologically plausible over a six-month horizon (Table 2). The Gompertz model’s asymmetric shape, implying a comparatively rapid deceleration after the inflection point followed by a long, slowly saturating tail, is consistent with the progressive but incomplete deceleration described in Section 3.2.1. This ranking describes in-sample fit to the 21-observation cumulative series and does not demonstrate that the best-fitting curve will provide reliable long-range forecasts (Section 3.5.2).

The Richards shape parameter was estimated at its lower bound (s = 0.01), so the fitted Richards curve is practically indistinguishable from the Gompertz curve (Figure 3). When this constraint was removed, the fit converged to a negative shape parameter (s ≈ −0.66) with a lower RMSE (18.9) but an upper asymptote exceeding 24,000 cases, indicating that final epidemic size is not identifiable from data through SitRep 61. The asymptotes K in Table 2 should therefore not be interpreted as estimates of final epidemic size.

#### 3.2.3. Interval-Level Growth-Rate and Doubling-Time Trajectory

**Figure 4:**
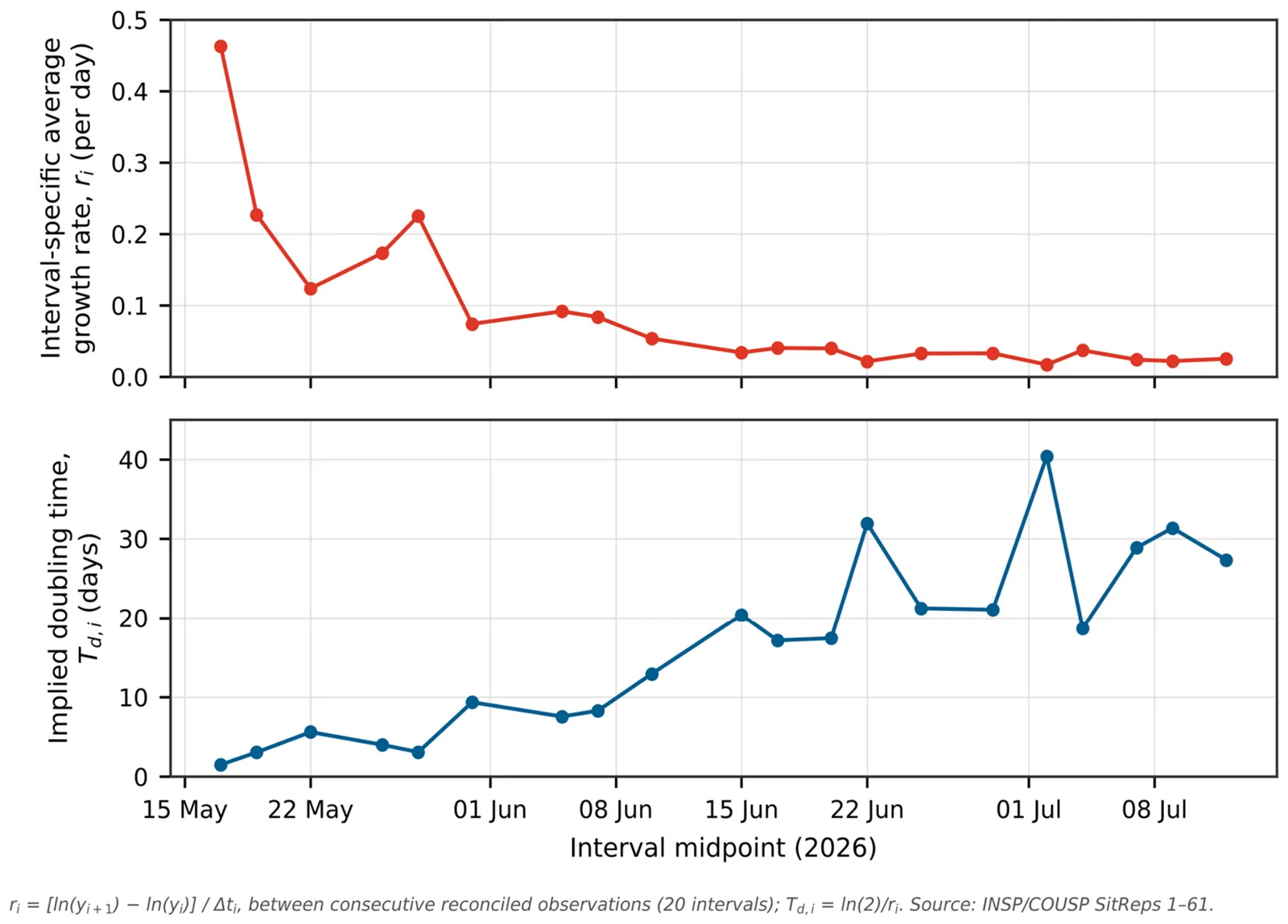
Interval-specific average proportional growth rate, *r_i_* (top), and implied doubling time, *T*_d,*i*_ (bottom), between consecutive reconciled observations (20 intervals), SitReps 1–61. Values are average rates over each interval, not instantaneous transmission rates; the underlying values are listed in Supplementary Table S1.

Interval-specific average growth rates and corresponding doubling times provided a finer-grained description of the changing reported trajectory (Figure 4; Supplementary Table S1). The largest rate, 0.463/day between SitReps 1 and 5, was strongly influenced by the initial count of eight cases and should not be interpreted as a stable transmission rate. Rates subsequently varied but generally declined, reaching 0.025/day in the final interval (SitReps 57–61). The absence of a negative interval rate by SitRep 61 indicates that the reported cumulative series had not plateaued or declined.

### 3.3. Geographic Spread

**Figure 5:**
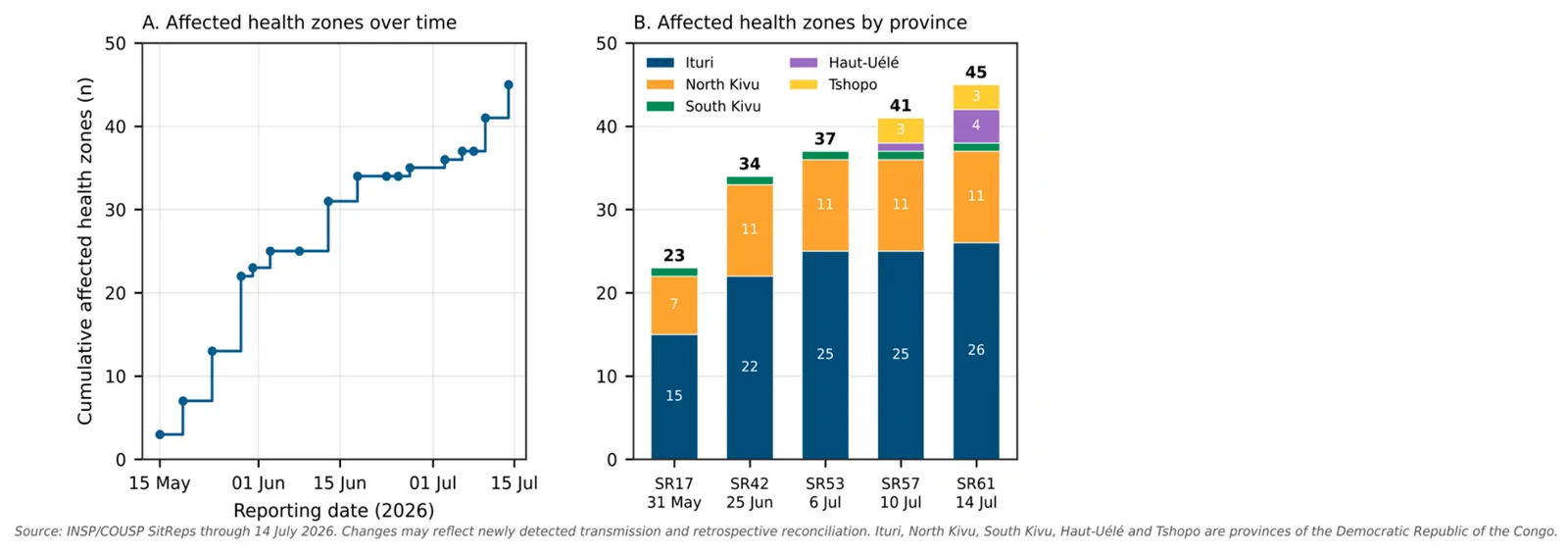
Geographic spread of the outbreak: (**A**) cumulative number of affected health zones by reporting date; (**B**) affected health zones by province at selected SitReps (17, 42, 53, 57 and 61). Ituri, North Kivu, South Kivu, Haut-Uélé and Tshopo are provinces of the Democratic Republic of the Congo. Source: INSP/COUSP.

The geographic footprint expanded substantially, increasing from 3 affected health zones in one province at SitRep 1 on 14 May 2026 to 45 health zones across five provinces by SitRep 61 on 14 July 2026, representing a 15-fold increase in the number of affected health zones (Figure 5).

The most rapid early expansion occurred during the second half of May, when the number of affected health zones rose from 7 on 19 May to 22 by 29 May. Geographic spread continued through June, reaching 35 health zones by 27 June and 36 by 30 June. During July, the outbreak expanded further with the recognition of additional affected zones and the formal inclusion of Haut-Uélé and Tshopo. By 14 July, affected areas included 26 health zones in Ituri, 11 in North Kivu, 1 in South Kivu, 4 in Haut-Uélé, and 3 in Tshopo.

Although the rate of newly affected health zones slowed relative to the initial expansion phase, geographic spread had not stabilised by mid-July.

### 3.4. Province-Level Comparisons

By SitRep 61 on 14 July 2026, Ituri accounted for 1863 of 2073 confirmed cases (89.9%) and 668 of 796 confirmed deaths (83.9%). North Kivu reported 189 cases and 111 deaths, corresponding to a reported CFR of 58.7%, compared with 35.9% in Ituri. These descriptive differences cannot distinguish variation in disease severity from outcome delay, preferential identification of severe or fatal cases, missed mild cases, access to diagnosis and care, or retrospective geographic reassignment.

**Figure 6:**
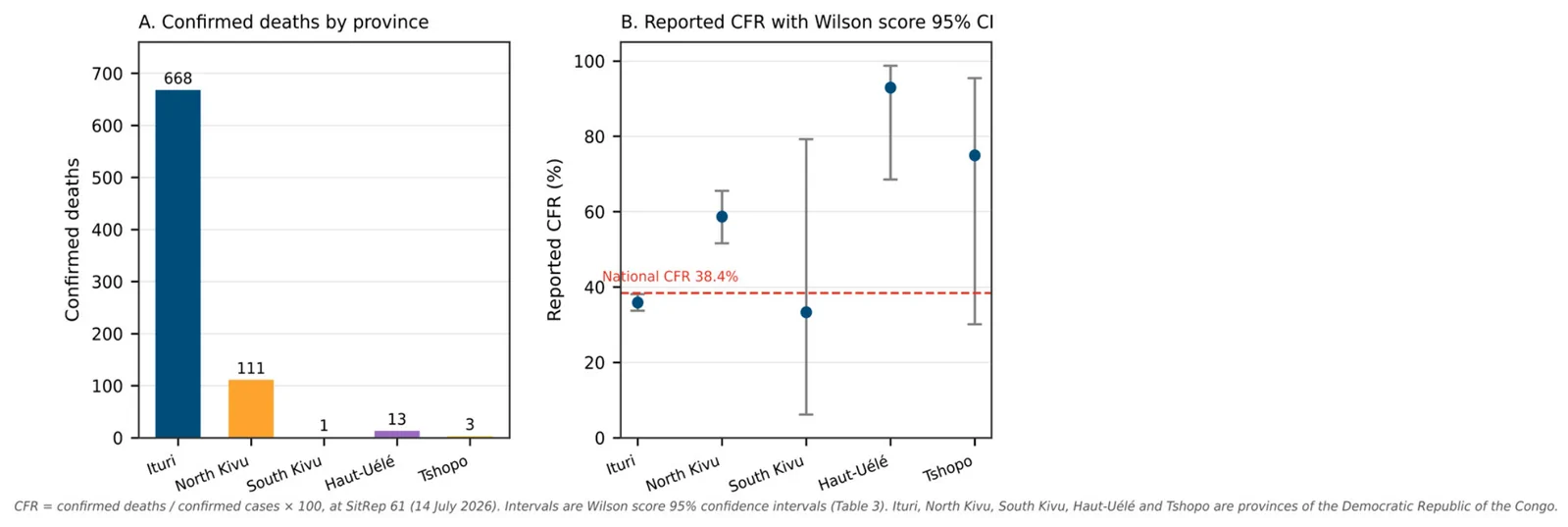
Confirmed deaths (**A**) and reported CFR with Wilson score 95% confidence intervals (**B**) for all five affected provinces at SitRep 61 (14 July 2026). All five provinces are shown so that the small-denominator estimates for South Kivu, Haut-Uélé and Tshopo can be read against their uncertainty; the dashed line marks the national CFR (38.4%). Ituri, North Kivu, South Kivu, Haut-Uélé and Tshopo are provinces of the Democratic Republic of the Congo.

The remaining provinces contributed relatively few cases. Haut-Uélé reported 14 cases and 13 deaths (CFR 92.9%), Tshopo 4 cases and 3 deaths (CFR 75.0%), and South Kivu 3 cases and 1 death (CFR 33.3%). These province-specific CFRs, particularly in Haut-Uélé and Tshopo, should be interpreted with considerable caution because they are based on very small denominators and are highly sensitive to the addition or reclassification of a single case or death. Wilson score 95% confidence intervals were calculated for each province’s CFR to make this denominator-driven imprecision explicit (Table 3; Figure 6): the intervals for Haut-Uélé (68.5–98.7%) and, especially, South Kivu (6.1–79.2%) are extremely wide, confirming that these point estimates alone should not be used to rank province-level severity. Overall, the provincial distribution at SitRep 61 indicates that the outbreak remained heavily concentrated in Ituri, while mortality was disproportionately high in North Kivu and in newly affected provinces with limited case counts.

### 3.5. Six-Month Scenario Projections

#### 3.5.1. Conditional Scenario Projections

**Figure 7:**
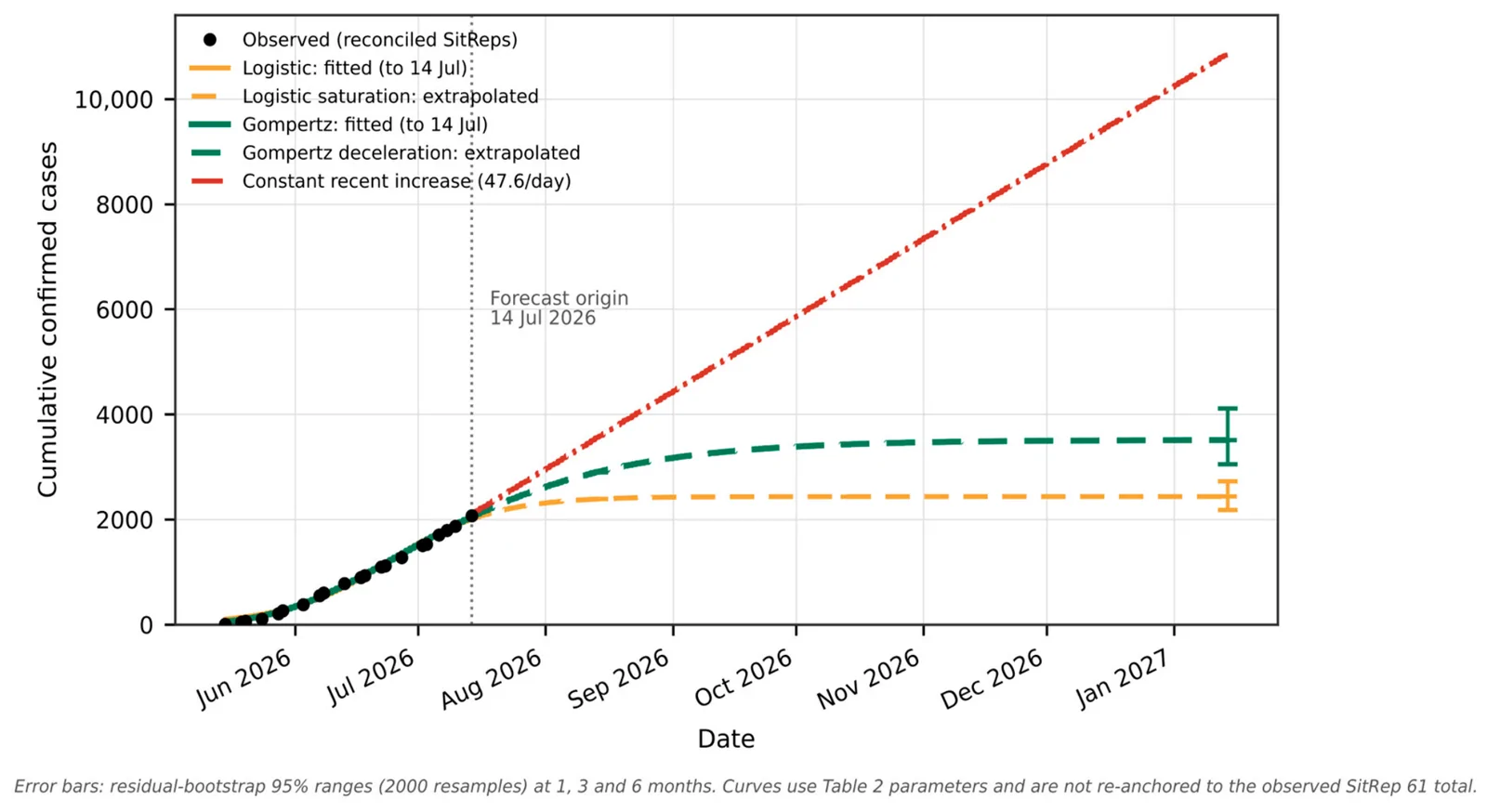
Six-month conditional scenarios for cumulative confirmed cases from the 14 July 2026 forecast origin. Black points: observed (21 reconciled SitReps); solid lines: in-sample fitted logistic and Gompertz curves (Table 2); dashed lines: extrapolated saturating scenarios; dash–dot line: constant recent-increase scenario (47.6 cases/day). Error bars show residual-bootstrap 95% ranges at 1, 3 and 6 months. The divergence between scenarios reflects structural uncertainty rather than a quantified effect of future interventions.

The three conditional scenarios diverged substantially after 14 July 2026 (Table 4; Figure 7). By 14 January 2027, the logistic saturation scenario reached approximately 2435 cumulative cases (residual-bootstrap 95% range 2182–2722), the Gompertz deceleration scenario approximately 3508 (3049–4110), and the constant recent-increase scenario approximately 10,839. Both saturating scenarios remain below their asymptotes (K = 2434.85 and 3511.99; Table 2). Their fitted values at the forecast origin (1992 and 2024 cases, respectively) were already below the observed SitRep 61 total of 2073, consistent with the underprediction observed in the temporal checks (Section 3.5.2). These values are conditional planning scenarios rather than calibrated predictions of final epidemic size. The long forecast horizon relative to the observation period and the use of serially correlated cumulative counts create substantial structural uncertainty.

#### 3.5.2. Limited Temporal Validation

In the limited temporal validation, Gompertz projections made using data through SitReps 39, 44, 50, and 55 under-predicted the subsequently reported SitRep 61 total by 27.0%, 21.9%, 12.2%, and 5.6%, respectively (Table 5). The error narrowed as later observations accumulated, but all four projections were below the reported total. These four checks share a single target date and overlapping training data, so they are not independent and do not establish a general pattern of systematic underprediction. Because only four origins were evaluated, these results should not be generalised as comprehensive forecast calibration.

## 4. Discussion

The reported cumulative trajectory showed slower proportional growth over time, but cases, deaths, and geographic extent continued to increase through 14 July 2026. This combination is consistent with epidemic deceleration without evidence of a plateau in reported cumulative cases. The aggregated analysis cannot determine why growth slowed. Possible explanations include response activities, changing surveillance or ascertainment, reporting delays and retrospective revisions, spatially heterogeneous local trajectories, and susceptible depletion within heavily affected populations. The available data do not permit these explanations to be separated or quantified.

Two structural factors may have contributed to the rapid early expansion: delayed recognition and laboratory confirmation of the outbreak, and the absence of licensed BDBV-specific vaccines and therapeutics, which limited the applicability of the ring-vaccination strategies deployed during the 2018–2020 North Kivu *Zaire ebolavirus* outbreak [20,21]. The aggregated data analyzed here cannot quantify the contribution of either factor.

The reported WHO declaration of a Public Health Emergency of International Concern shortly after the national outbreak declaration reflected concern regarding rapid geographic spread, the humanitarian context of eastern DRC, and the limited availability of species-specific medical countermeasures [22]. In this setting, clinical management depended largely on supportive care, while investigational products such as MBP134/MBP431, obeldesivir, and remdesivir required evaluation within appropriately regulated emergency-use or adaptive clinical-trial frameworks. These constraints highlight the comparatively limited clinical and operational evidence base available for BDBV relative to *Zaire ebolavirus*.

Reported CFRs varied substantially across provinces. North Kivu had a CFR of 58.7% (Wilson 95% CI 51.6–65.5%), compared with 35.9% in Ituri (95% CI 33.7–38.1%). This variation is unlikely to be explained by the natural history of Bundibugyo virus alone: the reported case fatality ratio during the 2007 Bundibugyo outbreak in western Uganda was approximately 32%, and long-term follow-up of that cohort documented substantial post-recovery morbidity [23,24]. These intervals describe uncertainty in each reported proportion but do not constitute a formal test of the between-province difference. The observed variation may reflect outcome delay, incomplete ascertainment, preferential identification of severe or fatal cases, missed mild cases, access to diagnosis and care, community deaths, or retrospective reclassification rather than biological differences in severity. Estimates for provinces with few reported cases are particularly unstable and should not be used to rank provincial disease severity.

Comparison with the 2014–2015 West African Ebola epidemic requires caution. That epidemic was caused by *Zaire ebolavirus*, and involved multiple countries and substantially different surveillance, intervention, and reporting contexts, whereas the present analysis concerns Bundibugyo virus disease and a 61-day national reporting window in the DRC. Published experience from 2014 also shows that real-time Ebola forecasts can change markedly as ascertainment and control conditions evolve [25,26]. Accordingly, a simple comparison of cumulative case totals over the first three calendar months could be misleading unless onset definitions, geographic scope, and reporting completeness are harmonised. The present outbreak should therefore be interpreted on its own transmission and operational trajectory rather than ranked directly against 2014 by crude case counts alone.

Growth-rate and doubling-time estimates derived from cumulative SitRep observations were serially correlated and vulnerable to reporting delay, changing ascertainment, and retrospective revision [25,26]. Their decline is compatible with epidemic deceleration but does not establish falling incidence, reduced transmission, or the effect of any specific intervention. Susceptible depletion is another possible contributor, but the data contain neither population-at-risk denominators nor local attack rates needed to evaluate it. These limitations require growth indicators to be interpreted alongside incident cases and surveillance and operational measures when such data are available.

The international dimension of the outbreak highlighted gaps in preparedness for non-Zaire orthoebolaviruses. Early Bundibugyo virus disease is clinically non-specific and overlaps with common febrile illnesses [27], so point-of-entry screening should emphasise epidemiological linkage, exposure history, longitudinal symptom monitoring, and rapid access to molecular confirmation rather than reliance on haemorrhagic manifestations alone. International situation assessments issued during the reporting period also documented cases in neighbouring Uganda and underlined the need for cross-border surveillance and coordination [22,28].

Community engagement, culturally appropriate risk communication, and accepted safe-burial practices have been identified as core rather than ancillary response functions in settings affected by displacement, insecurity, and mistrust; reporting community participation, refusal rates, and risk-perception indicators alongside epidemiological metrics would support adaptive outbreak management [29]. Clinical-trial readiness and equitable research partnerships have been identified as parallel priorities for this outbreak [30]. Neither dimension can be evaluated from the aggregated SitRep indicators analysed here.

By 14 July 2026, the provincial distribution remained highly asymmetric. Ituri accounted for 1863 confirmed cases across 26 health zones, North Kivu reported 189 cases across 11 health zones, and South Kivu recorded 3 cases in one health zone. Haut-Uélé and Tshopo contributed relatively few cases but reported very high CFRs. These contrasts may reflect differences in transmission intensity, population mobility, surveillance sensitivity, diagnostic access, health-seeking behaviour, and retrospective geographic reassignment. Because provincial contact-follow-up indicators were not consistently reported across all SitReps, comparisons between provinces should be made cautiously and should not be interpreted as direct measures of response effectiveness [31,32].

### 4.1. Model Comparison, Growth-Rate Reduction, and Forecast Reliability

The in-sample comparison favoured Gompertz and Richards curves over logistic and exponential curves [14,15], but good fit to an aggregated cumulative series does not ensure accurate long-range forecasts. In four temporal checks, Gompertz projections issued at earlier truncation points underpredicted the subsequently reported SitRep 61 total by 5.6–27.0%. The better in-sample fit of the Gompertz and Richards curves therefore does not establish long-range forecast accuracy. The consistent direction of error in these four checks, together with the short observation window and sensitivity of saturation models to recent data, indicates substantial structural uncertainty. The projection values should therefore be used as contingent, highly uncertain planning scenarios, not as upper bounds or deterministic forecasts [16,17].

Because the prespecified analytical dataset ended on 14 July 2026, case totals reported subsequently in August were not incorporated into model fitting or retrospective recalibration in this revision. They provide an important external validation opportunity: if later observed totals substantially exceed the July-based saturation scenarios, this would reinforce the evidence of structural under-prediction already seen in the four temporal checks. A formal update should use the complete post-14 July SitRep series rather than a single end-of-August count.

### 4.2. Limitations and Future Research

The analyses were restricted to national and province-level aggregates because the SitReps did not provide health-zone-by-date case series. A national curve can combine declining transmission in some locations with expanding transmission elsewhere. It therefore cannot determine whether deceleration was geographically uniform or driven by changing contributions from local outbreaks. Published accounts of the 2018–2020 North Kivu/Ituri epidemic describe transmission as spatially heterogeneous, with resurgences in particular health zones linked to insecurity and interrupted response access [33,34]. A single national growth-model fit averages over exactly this structure.

The same gap limits covariate analysis. Forest cover has been linked to Ebola spillover location [35]; road infrastructure and mobility have shaped this outbreak’s spatial trajectory [36]; and healthcare accessibility in Nord-Kivu is markedly uneven [37]. Testing these associations against growth rate, doubling time or final size requires health-zone-indexed case series and matched geospatial covariates, which the aggregated data cannot provide; we report this as open rather than approximate it. Trajectory-clustering of epidemic types [38] and daily-resolution early-warning indicators (acceleration, autocorrelation, spatial synchrony) [39,40,41] face the same constraint.

We regard health-zone-level line-list acquisition as the highest-priority extension, since disaggregated growth-model comparison, covariate analysis and clustering all follow directly from it; intervention-timing counterfactuals [42,43] are a natural subsequent step once that baseline exists.

Several limitations constrain this analysis. The study used aggregated, serially correlated cumulative surveillance counts that were affected by missing reports, reporting delays, incomplete ascertainment, changing surveillance sensitivity, retrospective reconciliation, and changing case classification. Eight sequentially numbered SitReps were unavailable, and the reasons for their unavailability were not documented in the extracted series. The analytical series comprised 21 of the 53 available SitReps, formed as described in the Reconciled Analytical Series and Data Lineage subsection rather than by a pre-specified sampling rule; fitting to all available reports could alter parameter estimates, although the observations used are provided in Supplementary Table S1 to allow for verification. The magnitude of historical data revisions could not be quantified because a revision log was unavailable. Reported CFRs may be biased by unresolved outcomes and preferential ascertainment of severe or fatal cases. Consistent denominators were unavailable for demographic missingness, preventing formal demographic or risk-factor analyses. The growth curves do not model intervention timing, spatial heterogeneity, or mechanistic transmission, and the limited four-origin temporal validation does not provide comprehensive forecast calibration. These constraints support cautious interpretation of the observed deceleration and conditional projections.

## 5. Conclusions

Reported cumulative cases, deaths, and geographic extent continued to increase through 14 July 2026 despite a general decline in interval-specific proportional growth. Gompertz and Richards curves described the observed cumulative trajectory better than logistic and exponential curves, but this in-sample advantage does not establish forecast accuracy, and Gompertz projections underpredicted the subsequently reported total in four temporal checks. The six-month values are therefore contingent, highly uncertain planning scenarios rather than reliable estimates of final epidemic size. Aggregated SitRep data cannot establish the causes of slowing, compare intervention effectiveness, or resolve whether provincial CFR differences reflect severity or ascertainment. Continued collection of dated incident cases, revision histories, outcomes, demographic variables, intervention timing, and health-zone-level data is necessary for more reliable transmission and forecast analyses.

## Figures and Tables

**Table 1:** Extended Epidemic Trajectory: Confirmed Cases, Deaths, CFR, and Geographic Spread, for 14 May–14 July 2026 (INSP Situation Reports 1–61).

SitRep Group	Final SitRep Used	Cum. Cases	Cum. Deaths	CFR (%)	HZ (n)	Provinces	Interval r (/Day)	Interval *T*_d_ (Days)
SR1–SR5	SR5	51	4	7.8	7	2	0.463	1.5
SR6–SR10	SR10	105	10	9.5	13	3	0.144	4.8
SR11–SR15	SR15	263	42	16.0	22	3	0.184	3.8
SR16–SR20	SR20	381	64	16.8	25	3	0.074	9.4
SR21–SR25	SR25	598	115	19.2	25	3	0.090	7.7
SR26–SR30	SR30	782	181	23.1	31	3	0.054	12.9
SR31–SR35	SR35	933	245	26.3	34	3	0.035	19.6
SR36–SR40	SR40	1118	291	26.0	34	3	0.036	19.2
SR41–SR45	SR44	1274	360	28.3	35	3	0.033	21.2
SR46–SR50	SR50	1528	492	32.2	36	3	0.030	22.9
SR51–SR55	SR55	1792	625	34.9	37	3	0.032	21.7
SR56–SR61	SR61	2073	796	38.4	45	5	0.024	28.6
Final situation	SR61	2073	796	38.4	45	5	—	—

Note: Cumulative indicators represent the value reported in the final available SitRep of each group and are not summed across SitReps. Interval growth rates and doubling times (columns 8–9) were computed between consecutive rows of this table (the first interval starts at SitRep 1, 8 cases), with Δ*t* equal to the difference in SitRep number. They are therefore coarser than the 20 intervals derived from the full 21-observation series (the Reconciled Analytical Series and Data Lineage subsection), which are reported in Section 3.2.3 and Supplementary Table S1. Rates in this table are computed between consecutive table rows and therefore differ slightly from the finer interval rates in Section 3.2.3. HZ = health zone; r = average daily proportional growth rate; *T*_d_ = doubling time.

**Table 2:** National-Level Growth Model Comparison, Fitted to 21 Reconciled Aggregated Case Observations (SitReps 1–61).

Model	Fitted Parameters	RMSE	AICc	BIC	Rank
Exponential	a = 209.45, b = 0.0391/day	127.97	270.78	272.50	4
Logistic	K = 2434.85, r = 0.0780/day, *t*_0_ = 41.73	55.94	239.11	240.79	3
Gompertz	K = 3511.99, b = 4.3745, r = 0.0340/day	34.14	218.37	220.05	1 (best)
Richards (generalised logistic)	K = 3485.76, r = 0.0344/day, *t*_0_ = 43.37, s = 0.010 (lower bound)	34.40	222.20	223.42	2

Note: Fitted at the national level only. Disaggregating this comparison by province or health zone would require case time series indexed by health zone and date that were not available in the aggregated SitRep summaries analysed here (Section 4.2). Time index t = SitRep number (t = 61 on 14 July 2026). AICc and BIC were computed from the Gaussian log-likelihood with the residual variance counted as a parameter. The Richards shape parameter was constrained to s ≥ 0.01; the estimate lies on this bound.

**Table 3:** Province-Level Ebola Burden Summary—SitRep 61, 14 July 2026 (with Wilson score 95% confidence intervals for CFR).

Province	Confirmed Cases	Confirmed Deaths	CFR (%)	Wilson 95% CI (%)	Affected HZ
Ituri	1863	668	35.9	33.7–38.1	26
North Kivu	189	111	58.7	51.6–65.5	11
South Kivu	3	1	33.3	6.1–79.2	1
Haut-Uélé	14	13	92.9	68.5–98.7	4
Tshopo	4	3	75.0	30.1–95.4	3
National total	2073	796	38.4	36.3–40.5	45

Note: CFR = confirmed deaths/confirmed cases × 100. HZ = health zone. 95% CI computed using the Wilson score method [13]. Provincial counts sum to the national totals.

**Table 4:** Conditional cumulative-case scenarios from the 14 July 2026 forecast origin (t = 61).

Scenario	Model	Basis	1 Month	3 Months	6 Months	6-Month Uncertainty
Logistic saturation	Logistic (Table 2)	Residual bootstrap 95% CI	2388	2434	2435	2182–2722
Gompertz deceleration	Gompertz (Table 2)	Residual bootstrap 95% CI	2898	3428	3508	3049–4110
Constant recent increase	Constant 47.6 cases/day	Not model-based	3550	6456	10,839	Not model-based

Note: Horizons are 1 month (14 August 2026; t = 92), 3 months (14 October 2026; t = 153) and 6 months (14 January 2027; t = 245). Logistic and Gompertz values are the Table 2 fitted curves evaluated at each horizon (fitted values at t = 61: 1992 and 2024, respectively), without refitting or re-anchoring; the uncertainty column gives percentiles of 2000 residual-bootstrap refits at 6 months. The constant recent-increase scenario extrapolates the mean reported absolute increase between SitRep 47 (1406 cases, 30 June) and SitRep 61 (2073 cases, 14 July): (2073 − 1406)/14 days = 47.6 cases per day. The numerical ranges of the scenarios are not directly comparable measures of probabilistic forecast uncertainty.

**Table 5:** Limited temporal validation of Gompertz projections for SitRep 61 using four earlier truncation points.

Truncation Origin	N Points Used	Gompertz Projection for SitRep 61	Actual SitRep 61 Value	Error (%)
≤SitRep 39	13	1514	2073	−27.0%
≤SitRep 44	15	1620	2073	−21.9%
≤SitRep 50	17	1820	2073	−12.2%
≤SitRep 55	19	1956	2073	−5.6%

## Data Availability

All data were derived from the publicly available official SitReps cited in this manuscript and from the referenced WHO and United Nations reports. The reconciled 21-observation analytical series, with reporting dates and source SitRep numbers, is provided in Supplementary Table S1. The code used for the retained growth-model comparison, interval-specific growth calculations, scenario projections, and limited temporal validation are available from the corresponding author on reasonable request.

## Supplementary Materials

The following supporting information can be downloaded at: https://www.mdpi.com/article/10.3390/v18101104/s1. Table S1. Reconciled analytical series of cumulative confirmed Bundibugyo virus disease cases used for interval-specific growth-rate estimation and growth-model fitting (21 observations; INSP/COUSP SitReps 1–61, 14 May–14 July 2026).
