# Supplementary material for: Rapid Geographic Expansion, Decelerating Growth, and Six-Month Scenario Projections of Bundibugyo Virus Disease in the Democratic Republic of the Congo: Analysis of Situation Reports 1–61
Source: Viruses. 2026 Oct 6;18(10):1104. doi: 10.3390/v18101104 (PMC13709745; doi:10.3390/v18101104)
Supplement: Supplementary file 1 [file viruses-18-01104-s001.zip › viruses-4596409-supplementary.pdf]

Supplementary Materials

Table S1. Reconciled analytical series of cumulative confirmed Bundibugyo virus disease cases used for interval-specific growth-rate estimation and growth-model fitting (21 observations; INSP/COUSP SitReps 1–61, 14 May–14 July 2026).

Obs.	Source SitRep	Reporting date	t	Cumulative confirmed cases	Cumulative confirmed deaths[1]	Table 1	Figure 2 label	Interval (SitReps)	Δt (days)	r_i (/day)	T_d,i (days)
1	SR1	14 May 2026	1	8	4	Yes	Yes	—	—	—	—
2	SR5	19 May 2026	5	51	4	Yes	No	1–5	4	0.463	1.5
3	SR6	20 May 2026	6	64	—	No	Yes	5–6	1	0.227	3.1
4	SR10	24 May 2026	10	105	10	Yes	Yes	6–10	4	0.124	5.6
5	SR14	28 May 2026	14	210	—	No	Yes	10–14	4	0.173	4.0
6	SR15	29 May 2026	15	263	42	Yes	No	14–15	1	0.225	3.1
7	SR20	3 June 2026	20	381	64	Yes	Yes	15–20	5	0.074	9.4
8	SR24	7 June 2026	24	550	—	No	Yes	20–24	4	0.092	7.6
9	SR25	8 June 2026	25	598	115	Yes	No	24–25	1	0.084	8.3
10	SR30	13 June 2026	30	782	181	Yes	Yes	25–30	5	0.054	12.9
11	SR34	17 June 2026	34	896	—	No	Yes	30–34	4	0.034	20.4
12	SR35	18 June 2026	35	933	245	Yes	No	34–35	1	0.040	17.1
13	SR39	22 June 2026	39	1,094	—	No	Yes	35–39	4	0.040	17.4
14	SR40	23 June 2026	40	1,118	291	Yes	No	39–40	1	0.022	31.9
15	SR44	27 June 2026	44	1,274	360	Yes	Yes	40–44	4	0.033	21.2
16	SR49	2 July 2026	49	1,502	—	No	Yes	44–49	5	0.033	21.1
17	SR50	3 July 2026	50	1,528	492	Yes	No	49–50	1	0.017	40.4
18	SR53	6 July 2026	53	1,708	—	No	Yes	50–53	3	0.037	18.7
19	SR55	8 July 2026	55	1,792	625	Yes	No	53–55	2	0.024	28.9
20	SR57	10 July 2026	57	1,873	—	No	Yes	55–57	2	0.022	31.4
21	SR61	14 July 2026	61	2,073	796	Yes	Yes	57–61	4	0.025	27.3

Notes. t = time index used in all model fits (t = SitRep number; t = 61 on 14 July 2026). Values are cumulative totals as published in each SitRep; retrospective corrections in later reports were not back-propagated. "Table 1" and "Figure 2 label" indicate whether the observation appears in manuscript Table 1 and/or is annotated in Figure 2; the analytical series is the union of these two sets (see Section 2.3.1). $r_i = [\ln(y_{i+1}) - \ln(y_i)]/\Delta t_i$, computed between the listed observation and the preceding one; $T\_d,i = \ln(2)/r_i$. These 20 intervals are plotted in Figure 4. Fitting

the exponential, logistic, Gompertz and Richards ($s \geq 0.01$) models to these 21 observations with this time index reproduces the parameter estimates in Table 2; refitting the Gompertz model to observations up to SitReps 39, 44, 50 and 55 (13, 15, 17 and 19 observations) reproduces Table 5.

[1] Cumulative confirmed deaths are shown only for the 13 SitReps reported in Table 1; deaths were not used in model fitting.
